# Bacterial outer membrane vesicles, a potential vaccine candidate in interactions with host cells based

**DOI:** 10.1186/s13000-018-0768-y

**Published:** 2018-12-11

**Authors:** Wei Cai, Dinesh Kumar Kesavan, Jie Wan, Mohamed Hamed Abdelaziz, Zhaoliang Su, Huaxi Xu

**Affiliations:** 1 Department of Immunology, Jiangsu University, Zhenjiang, 212013 Jiangsu China; 2 The Central Laboratory, the Fourth Affiliated of Jiangsu University, Zhenjiang, 212001 China

**Keywords:** Outer membrane vesicles, Host cells, Vaccines, Bacterium, Immunity

## Abstract

Both Gram-Positive and Gram-Negative bacteria can secrete outer membrane vesicles (OMVs) in their growth and metabolism process. Originally, OMVs were considered as a by-product of bacterial merisis. However, many scientists have reported the important role of OMVs in many fields recently. In this review, we briefly introduce OMVs biological functions and then summarize the findings about the OMVs interactions with host cells. At last, we will make an expectation about the prospects of the application of OMVs as vaccines.

## Background

**Fig. 1:**
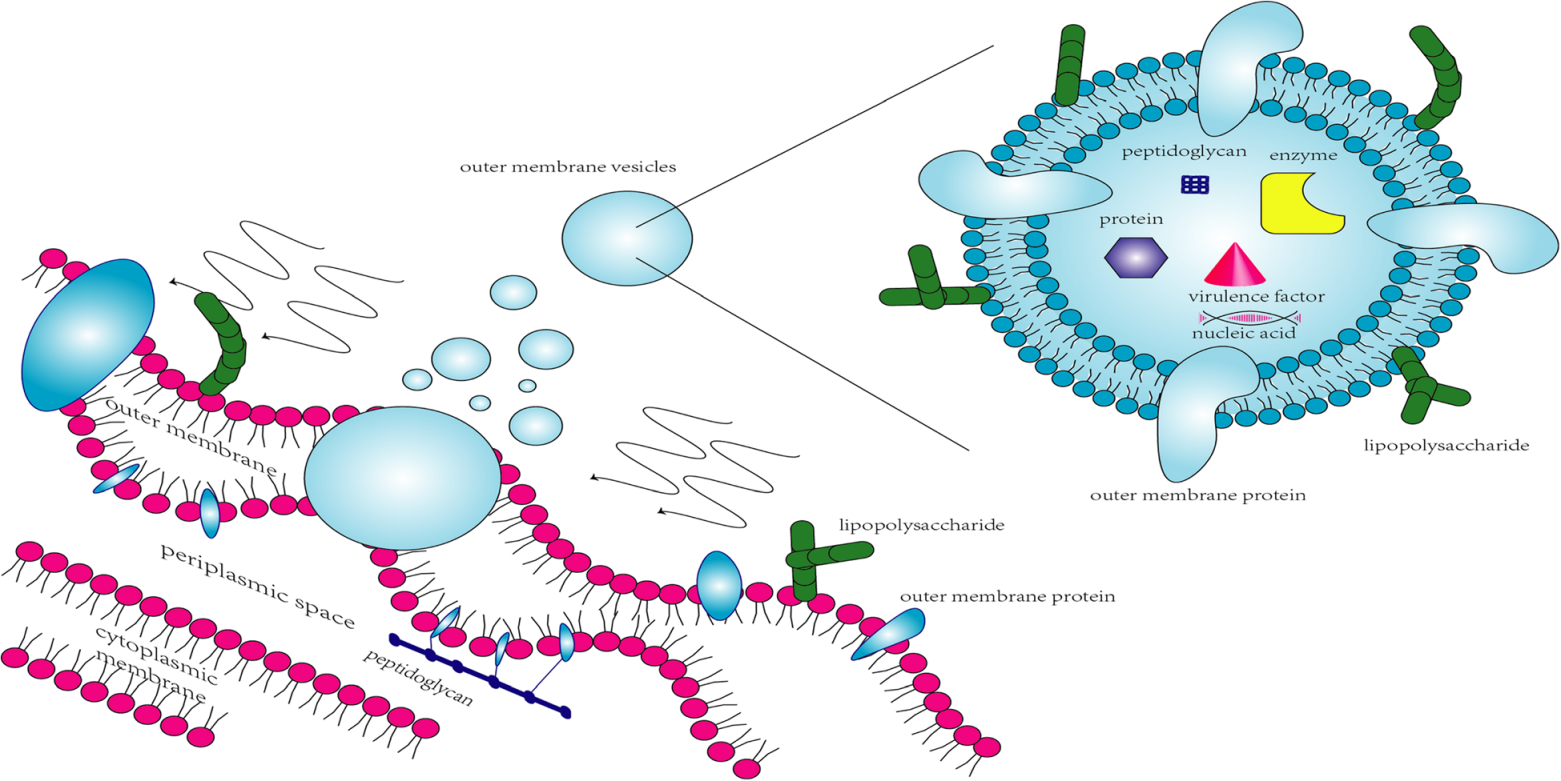
Visualization of the outer membrane vesicles about structures and components. Legend section: nucleic acid; virulence factor; protein; enzyme; peptidoglycan; outer membrane protein; lipopolysaccharide; outer membrane vesicles; outer membrane; periplasmic space; cytoplasmic membrane

OMVs secretion is a normal phenomenon during the growth of bacteria. OMVs are spherical, bilayered, membranous nanostructures (shown in Fig. 1). OMVs productions are not consistent in size, varying from approximately 20 nm to 250 nm. The parent bacterial membrane releases them and hence they contain numerous proteins, similar to parent bacteria [1–4]. Not only that, OMVs are demonstrated in abundance in bacterial components such as DNA [5], RNA [6, 7], lipopolysaccharide (LPS) [8], enzymes [9, 10], peptidoglycan [11] and some molecules [10]. Here, we enumerate some examples about virulence factors carried by OMVs (listed in Table 1). These characteristics of OMVs endow with critical significance in pathogenesis and communication between bacteria and host cells. Recent studies have revealed that OMVs play fundamental roles in activating immune system and facilitate manifest responses against OMVs in the host [12]. Despite of the increasing findings about effects of OMVs on host, innovative strategies based on OMVs have shown great potential in clinical implications and other relevant fields as we will discuss in this review.

**Table 1:** Important virulence factors found in bacterial outer membrane vesicles

Parent bacterium	Classification	Virulence factors	Description	Putative functions	References
*Pasteurella multocida*	Gram negative	β-lactamase (only found in Pm12945) OmpA, OmpH, OmpW, Tbp	Enzymes Proteins DNA	Disease pathogenesis, Deliver drug- resistant gene	16
Myxococcus xanthu	Gram negative	MepA, Several molecules with antibiotic properties, Hydrolytic enzymes	Proteins Enzymes	Hydrolytic function, antibiotic activities	17
*Bacteroides fragilis*	Gram negative	B.fragilis toxin (BFT).	protease	toxin packaging and delivery	18
Vibrio cholerae	Gram negative	VrrA, OmpA	RNA, Proteins	OMVs regulation	19
Bacillus subtilis	Gram positive	Lipoproteins, siderophore-binding proteins	Proteins	biosynthesis	20
Enterohemorrhagic *Escherichia coli* (EHEC)	Gram negative	Shiga toxin 2a, cytolethal distending toxin V, EHEC hemolysin, flagellin	Proteins	cell cycle arrest and pathogenesis	21

## Simplified introduction of OMVs biological functions and biosynthesis

OMVs are not described useless as originally thought. Electron microscopy [2–4, 10] and proteomic analyses [1–4] have confirmed OMVs are heterogeneous nanostructures packaging various bioactive components. They function as mediators to transmit biological information among different bacteria and host. For example, bacterial OMVs serve as nanovesicles to deliver important biological substances, which can promote the entry of bacterial antigens or even genes. Colitogenic *Bacteroides thetaiotaomicron* OMVs assist bacterial antigens access host cells in a sulfatase-dependent manner [13]. Bacterial OMVs shoot short RNAs to boost the host-pathogen interaction [6, 7]. In addition, OMVs can act as a delivery system for virulence factors [14] or antibiotic resistence genes [15]. For instance, Bacteroides thetaiotaomicron OMVs carry cephalosporinases to protect gut pathogens against β-lactam antibiotics [16]. Consequently, considering OMVs as effective vehicles to enhance the interactions between host and pathogen, the biosynthesis of OMVs deserve prominent attention to make us better understanding of OMVs as we will discuss below.

Interestingly, although both Gram-positive and Gram-negative bacteria have the capacity of producing OMVs [17], the amounts of OMVs production and the components carried by OMVs are different with each other, even the same bacteria in different environment [18, 19]. Also, heterogeneous sized OMVs contain distinct protein profiles [20].Usually, it is considered that OMVs production as responses to stress around bacteria to make bacteria survive and adapt within host [18]. OMVs released from *Vibrio cholerae* appear heterogenous in size due to different culture environment and purification methods that affect chemical composition of OMVs as well [21]. Ciprofloxacin-stimulated cultures produced more and larger vesicles which were enriched with cytosolic proteins compared with non-induced condition [22]. Not just ciprofloxacin, antibiotic such as meropenem, fosfomycin, and polymyxin B can also increase production of OMVs [23]. Furthermore, amounts of OMVs produced by cultured clean water bacteria increased upon treatmentwith ultraviolet radiation [24]. *Pseudomonas putida* secrete OMVs in response to stress caused by cationic surfactants [25]. There is a hypothesis that bacteria produce OMVs as defense mechanisms against external threats, including antibiotics [26], antimicrobial peptides [27], and bacteriophage infection [28], which may enlighten us of the important role of OMVs in bacterial pathopoiesis. There are evidences indicating that *Porphyromonas gingivalis* (*P. gingivalis*) OMVs contribute to local immune evasion of *P. gingivalis* by hindering the host response [29].

With the exception of the findings discussed above, many factors may be responsible for OMVs biosynthesis according to recent study: (1) Loss of some antigens of bacteria may contribute to proteins transformation in OMVs [30] and virulence factor may regulate OMVs biogenesis [31]. (2) Sandro et al., reported that the VacJ/Yrb ABC (ATP-binding cassette) transport system is involved in OMV formation among Gram-negative bacteria [32]. Pseudomonas Quinolone Signal (PQS) is found to modulate OMVs production in *Pseudomonas aeruginosa* [33]. On the basis of the discovery PQS, Alexander et al.*,* demonstrated reciprocal cross-species can induce OMVs biogenesis via secreted factors in Gamma proteobacteria but not included Alphaproteobacteria [34]. (3) The formation of *Vibrio vulnificus* OMVs is associated with expression of the capsular polysaccharide [35]. Enterohemorrhagic *Escherichia coli* OmpT have an impact on the biogenesis, composition, and size of OMVs [36]. In addition, pathogenic or non-pathogenic bacterial OMVs may show great discrepancy in biological activity. Recent study reveals that toxigenic *Bacteroides fragilis* (*B. fragilis*) OMVs represent different metabolic activities compared with nonpathogenic *B. fragilis* OMVs [37]. In addition, emerging evidences indicate that close relationships between bacterial components and OMVs may modulate production of OMVs.Wael et al. declared that LPS remodeling leads to formation of OMVs in *Salmonella* [38]. Contrarily, *Salmonella* OMVs can also accelerate LPS remodeling during environmental transitions [39]. Haruyuki et al.*,* found if DNA inversion occur in *B. fragilis*, it will regulate the formation of OMVs [40], and small RNAs may modulate the composition of outer membrane proteins, which may be critical to OMVs synthesis [41]. In conclusion, these innovative findings have expanded our knowledge of how OMVs generate from parent bacteria. We can speculate from so many evidences about OMVs production that whether OMVs serve as a mirror reflecting the surrounding environment around bacteria and the real time condition of bacteria or not. If it is right, we can evaluate the level of bacterial condition through quantitative analysis of OMVs, which may be a promising idea concerning monitoring the growth of bacteria involved in related fields. However, the mechanisms of OMVs formation have not been explored clearly, no unanimous conclusion can be made in this field. It deserves more attention and more research to find the truth concealed behind the phenomenon.

## Interactions of bacterial OMVs with host cells

Despite the mystery on the mechanisms involved in OMVs formation, the research on the functions of bacterial OMVs especially with host cells keep growing rapidly. Next, we will focus on the correlation between bacterial OMVs and host cells, mainly including the OMVs entry mechanisms of host cells together with the effects of OMVs on host cells.

### How can host cells uptake OMVs?

Bacterial OMVs are recognized as external foreign matters by host cells. Considering OMVs carry numerous components of their parent bacteria, are they responsible for the entry of OMVs into host cells? It is widely accepted that several main pathways may promote the entry of OMVs including macropinocytosis [20], lipid raft-dependent or lipid raft-independent endocytosis, and clathrin- [42], caveolin- [20] and dynamin-dependent [43] entry (reviewed in REF47). Recent study may expand our knowledge of this topic. Sivapriya et al.*,* show clear evidences that bacterial OMVs deliver LPS into host cells via endocytosis, then LPS released from early endosomes into cytosol to induce the activation of caspase-11 and the secretion of inflammatory cytokines [44]. LPS structure especially the O antigen structural region is critical to OMVs entry. OMVs lacking O antigen may use clathrin-mediated endocytosis as a main route of entry nevertheless OMVs with intact O antigen are mediated by raft-dependent pathways [45]. *Helicobacter pylor* OMVs size can determine their mechanisms of host cells entry [20]. Moreover, bacterial OMVs express pathogen associated molecular patterns (PAMPs) on their surface. It may activate TLR signaling to facilitate OMVs entry into host cells. Lan et al.*,* argues that the activation of toll like receptor 4 (TLR4) contribute to OMVs deliver LPS into cytosol [46]. *Legionella pneumophila* OMVs membrane fuse with eukaryotic membrane systems, which may deliver pathogen factors to host cell membranes [47]. In brief, bacterial OMVs have their specific routes to host cells and the properties of OMVs may affect their decision about which road to choose when they asses into host cells. Anyway, OMVs can enter into host cells via different manners so that they can arouse strong response to cells as discussed below.

### Impressive effects of OMVs on host cells

Bacterial OMVs bleb from bacterial outer membrane, amounts of proteomics and chemical analysis show that they carry plenty of virulence factors coming from bacteria as we have discussed before [48–51]. Hence, OMVs inherit similar immunogenicity to parent bacteria. When OMVs are utilized to examine the reaction of host cells, meaningful results come out. Next, we will focus on the discussion about the OMVs interactions with host cells and review the recent study on the field.

**Fig. 2:**
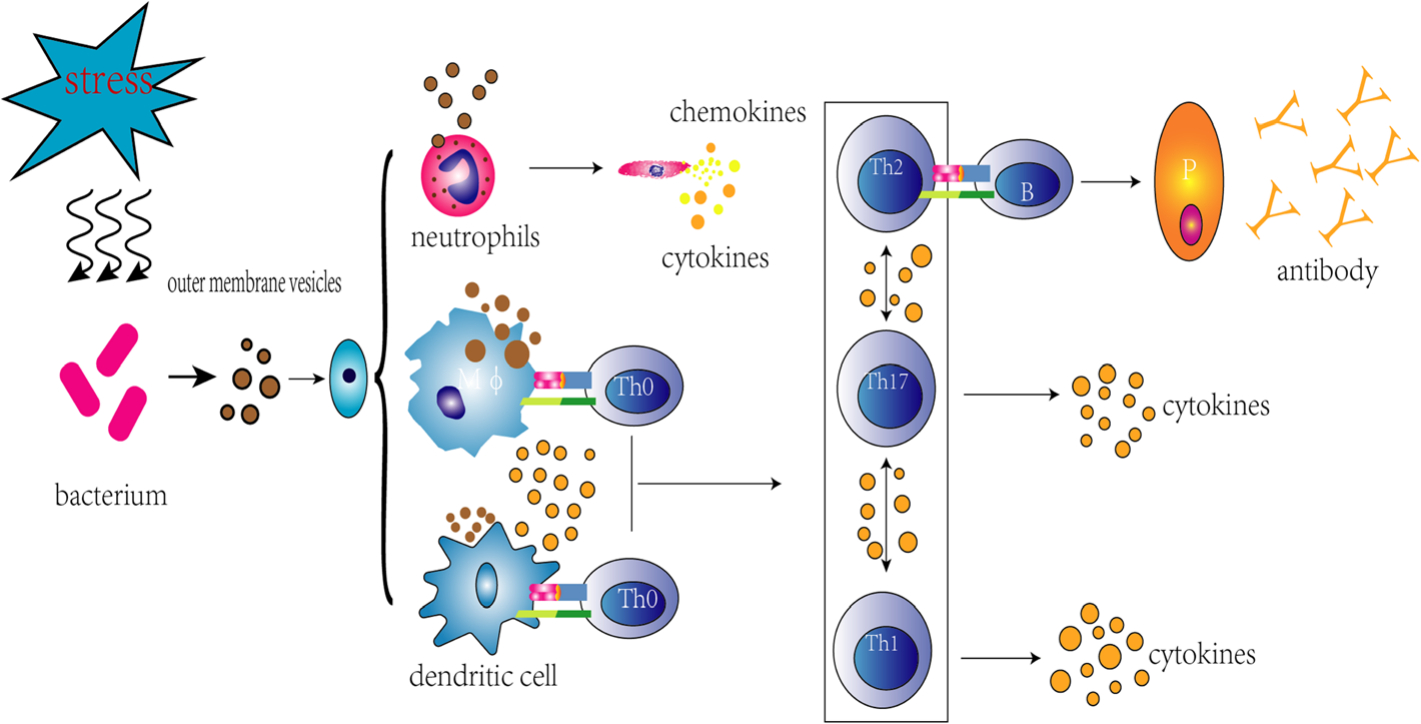
Brief illustration about interactions involved in OMVs and main immune cells. Legend section: stress; bacterium; outer membrane vesicles; neutrophils; dendritic cells; chemokines; cytokines; antibody

Usually, OMVs invade into host cells and bypass the epithelial cells barrier. Then, they will be exposed to innate immune cells mainly including neutrophils, macrophages and dendritic cells resident in submucosa and these immune cells will be activated to elict inflammatory responses against OMVs. In addition, adaptive immune cells involved in B cells and T cells will be awaked by signal molecules released from antigen presenting cells (APCs). In summary, the interactions between OMVs and immune cells (shown in Fig. 2) are manifested and this will be elucidated separately.

#### OMVs with neutrophils

Although OMVs are considered as non-duplicate, inanimate form nano structures, they can activate neutrophils to induce inflammatory cytokines in host. For example, *Neisseria meningitidis* associated OMVs simulate human neutrophils to produce pro-inflammatory profile of cytokines and chemokines including interleukin1-β (IL1-β), IL-8, tumor necrosis factor alpha (TNF-α), macrophage inflammatory protein 1α (MIP-1α), and MIP-1β [52]. Further study demonstrate gamma interferon (IFN-γ) can improve the level of these cytokines to maintain the condition of chronic inflammation [52]. These responses are similar to neutrophils triggered by bacterial infection, it appears that OMVs can contribute to protective immunity against infection. On the contrary, some bacterial virulence factors carried by OMVs can inhibit the antimicrobial activity of neutrophils and hence contribute to attenuate the secretion of cytokines simulated by OMVs. For example, uropathogenic *Escherichia coli* (UPEC) OMVs carry cytotoxic necrotizing factor type 1 (CNF1). CNF1 are recognized as toxin that can decrease the membrane fluidity of polymorphonuclear leukocytes (PMNs), which may contribute to functional impairment of PMNs and hence decreased profile of cytokines and chemokines [53]. Despite of the demonstration of OMVs effects on neutrophils, novel opinions appear that *Neisseria meningitidis* OMVs can be neutralized by plasma and bactericidal/permeability-increasing protein (BPI) and the neutrophils disable to clean the production of OMVs [54].

In contrast, pathogens are responsible for the death of neutrophils. When neutrophils try to prevent the invasion of bacteria, they will sacrifice themselves to trigger killing defense mechanism against pathogens. Neutrophil extracellular traps (NETs) are a novel bacterial killing mechanism that enable neutrophils clean pathogen rapidly [55]. Bacterial OMVs are reported that they can induce NETs formation as well [56]. However, *Neisseria meningitidis* can escape NETs involved innate response, which may increase the amounts of OMVs and promote the progression of infection [56]. We can summarize from the discussion above that neutrophils show great discrepancies in immune response to bacterial OMVs.

#### OMVs with macrophages

Macrophages act as a classical type of immune cells, they can elicit potent immune responses when they are treated with bacterial OMVs. Firstly, OMVs enforce macrophages to secrete pro-inflammatory cytokines. OMVs pretreatment evoked inflammatory responses in macrophages [3, 57–59]. Bacterial OMVs are phagocytosed by macrophages and activated macrophages then induce increased production of TNFα, IL-8, and IL-1βthrough NF-κB activation [60]. Macrophages simulated with *P. gingivalis* OMVs produce more TNFα, IL-12p70, IL-6, IL-10, IFNβ, and nitric oxide [61]. Isolated *Legionella pneumophila* (*L.pneumophila*) OMVs have the pro-inflammatory potential on macrophages dependent on TLR2/4 pathway [62]. Furthermore, OMVs facilitate *L. pneumophila* replication in macrophages. The finding may explain OMVs promote spreading of *L. pneumophila* in the host [63]. Guanylate Binding Proteins (GBP) are demonstrated as regulators of OMVs-mediated inflammation when using avirulent *Escherichia coli* OMVs to infect bone marrow-derived macrophages (BMDMs) [64]. However, porin loss OMVs may elicit lower levels of proinflammatory cytokine secretion compared with *Klebsiella pneumoniae* OMVs with porin [65]. Secondly, macrophages simulated by bacterial OMVs may cause adaptive immune response. *Neisseria meningitidis* [66] or *Klebsiella pneumoniae* [67] OMVs upregulate the expression of molecules supporting antigen presentation on the surface of macrophages, for example, cluster of differentiation 80(CD80), major histocompatibility complex-II (MHC-II), CD86 [59], human leukocyte antigen-DR (HLA-DR), and intercellular adhesion molecules-1(ICAM-1). As one of the professional antigens presenting cells, macrophages enable to simulate T cells to recognize OMVs antigens and then promote adaptive immune response. Naive peritoneal macrophages treated with *Shigella boydii* OMVs can trigger polarization of CD4+ T cells toward Th1 adaptive immune response [58]. Thirdly, bacterial OMVs can make macrophages metabolic remodeling and induce pyroptosis [61] and apoptosis [68] as reported. These effects on macrophages may contribute to decreased amounts and dysfunction of protective cells, which may be considered as an important factor leading to diseases in host body.

With the exception of inflammatory role in macrophages, bacterial OMVs can play anti-inflammatory roles in infected host cells. Previous findings have revealed that macrophages treated with OMVs will secrete more anti-inflammatory cytokines such as IL-10 [58, 61]. *Helicobacter pylori* OMVs lead to more production of immunosuppressive cytokine IL-10 by human peripheral blood mononuclear cells (PBMC) and apoptosis in Jurkat T cells [69]. Accordingly, it can be considered that on the one hand, bacterial OMVs elicit potent immune response against infection, and on the other hand, they benefit bacteria by limiting the inflammation and destroying immune cells to promote bacterial survival in the host. It seems like double-edged sword as to host hence we need to make best use of the advantages and bypass the disadvantages of OMVs by exploring effective instructions about them.

#### OMVs with dendritic cells

Similarly, bacterial OMVs can activate dendritic cells by upregulating the expression of co-stimulatory molecules and inducing the cytokines profile [70]. Meningococcal OMVs have the capacity of stimulating dendritic cells by inducing high expression of the CD40, Programmed death-ligand 1(PD-L1), CD83, CD80, CD86, and HLA-DR activation markers. Meanwhile, dendritic cells stimulated by Meningococcal OMVs produce more cytokines such as IL-6, IL1β compared with un-stimulated dendritic cells [71]. *Helicobacter pylori* OMVs induce dendritic cells to express more Heme Oxygenase-1 via activating Akt-Nrf2 and mTOR-κB Kinase–NF-κB pathways [72]. Collectively, exposure of dendritic cells to bacterial OMVs may trigger innate immune response against bacterial infection [73–79]. As professional APCs, the up-regulation of the co-stimulatory molecules expression have critical significance in triggering adaptive immune response as will be discussed next.

#### OMVs with B cells

It is well acknowledged that B cells mediate humoral immunity via mainly secreting antibody such as immunoglobulin (Ig) to protect host from pathogen infection. Usually, B cells require assistance of T cells to response to external antigen. *Salmonella typhimurium* OMVs activate prime B cells response together with T cells and the specific IgG can be detected in mice immunized with OMVs [59]. Conversely, OMVs can directly simulate B cells response without the help of T cells [80]. Furthermore, the activity of proliferation will be potentiated by OMVs simulation and the B cell receptor (BCR) may be a prerequisite for this mitogenic response [80]. *Neisseria lactamica* can induce the secretion of polyclonal IgM by B cell proliferation, resulting in colonizing and immune tolerance in host without adaptive immune response [81]. To explore the interaction between OMVs and B cells, a novel mechanism may explain the activation of B cells by bacterial OMVs. *Moraxella catarrhalis* produce OMVs to help them escape from host immune response [82]. The author clarifies the procedure of how B cells respond to OMVs. First, BCR internalization and then IgD BCR clustering and Ca^2+^ mobilization will be triggered on the membrane of activated B cells. Then, some motifs including IgD-binding super antigen MID, unmethylated CpG-DNA are critical for activation. At last, the production of IL-6 and IgM together with increased expression of surface marker (HLA-DR, CD45, CD64, and CD86) follow by activation CD19^+^ IgD^+^ lymphocytes [82]. In conclusion, OMVs have widespread effects on B cells as one important part of adaptive immune response.

#### OMVs with T cells

When OMVs gain access into host, APCs present OMVs antigen to CD4^+^T cell and then facilitate T helper cell (Th) differentiation into mainly three subtypes including Th1, Th2, Th17, which contribute to cell immune response together with humoral immune response. On the basis of the theory, OMVs show potential adjuvant effects on T cells cross-priming involved in CD4^+^T cell response and CD8^+^T cell response [83]. In brief, OMVs can elicit potent protective immune responses, which may make OMVs become effective vaccines candidate.

However, recent study shows that OMVs may suppress T cell response and proliferation via various manners. For example, *Neisseria meningitidis* OMVs carried Opa proteins could affect T cell proliferation by changing receptor binding [84]. Helicobacter pylori OMVs are demonstrated to inhibit T cell proliferation through inducing the COX-2 expression in monocytes [85]. In addition, *Neisseria gonorrhoeae* PorB presenting in OMVs can suppress CD4^+^T cell proliferation while PorB proteosomes can alter the immunosuppression [86]. The negative effects on immunity may contribute to bacterial survival and invasion, which should be highlighted to learn more about pathogenicity of bacteria.

#### OMVs with other host cells

Except the effects of OMVs on immune cells, numerous cells can respond to OMVs as we will discuss below. There are reports that bacterial OMVs can also induce morphological changes in host cells [87, 88]. Meanwhile, similar to the effects on immune cells, OMVs can induce inflammatory responses when they are exposed to host cells. *Stenotrophomonas maltophili* OMVs elicit a potent inflammatory response in human lung epithelial A549 cells [89]. *Vibrio cholerae* OMVs are responsible for inflammatory responses via secreting biologically active proteases [90]. *Pseudomonas aeruginosa* OMVs activate inflammasome through caspase-5 in human THP-1 monocytes [91]. *Escherichia coli* derived OMVs can elicit immune response [92] and promote interleukin 8 production in human intestinal epithelial cells [93]. However, the interactions are complicated and different between various OMVs and species. *Acinetobacter baumannii* OMVs show phospholipase, hemolytic and leucotoxic activities when treated with red blood cells and white blood cells [94]. *Helicobacter pylori* OMVs play an important role in eosinophil degranulation [95]. Bacterial OMVs- associated DNA can internalize by epithelial cells, which may suggest important role of OMVs in host-pathogen interactions [5]. *Aggregatibacter actinomycetemcomitans* OMVs are internalized in human embryonic kidney cells and trigger innate immune response [11]. *P. gingivalis* OMVs can promote calcification of vascular smooth muscle cells through ERK1/2-RUNX2 [96] and initiate innate immune responses of human endothelial cells [97]. Probiotic *Escherichia coli* OMVs contribute to the reinforcement of the epithelial barrier by regulating expression of tight junction proteins in intestinal epithelial cells [98].A similar strategy is utilized by *Escherichia coli* to strengthen the leukocyte binding on endothelial cells, as OMVs increase the expression of functional cell adhesion molecules [99]. On the contrary, *Pseudomonas aeruginosa* OMVs induced by human mucosal fluid and lysozyme can compromise an epithelial barrier [100].*Campylobacter jejuni* OMVs have proteolytic activity to clean intestinal epithelial cell E-cadherin and occludin [101]. We can draw a conclusion from the reports above that bacterial OMVs may be responsible for the interactions of pathogen with intestinal epithelial cells.

#### OMVs associated with apoptosis in host cells

Despite of bonus bring from OMVs, OMVs shed from bacteria can directly induce host cells apoptosis due to the virulence factors inside OMVs [4]. Host cells treated with EHEC O157 OMVs develop G2 cell cycle arrest and the study proves OMVs can serve as carriers of EHEC O157-mediated host injury [102]. At the same time, OMVs associated with the probiotic *Escherichia coli* lead to DNA damage in intestinal epithelial cells [42]. The outbreak strain *Escherichia coli* O104:H4 derived OMVs carry virulence factors will cause apoptosis of human intestinal epithelial cells [43]. OMVs from *Neisseria gonorrhoeae* target PorB to mitochondria and induce apoptosis [6]. *Acinetobacter baumannii* omp33–36 porin induces apoptosis and modulates autophagy in *HeLa* and HEp-2 cells [103]. *Acinetobacter nosocomialis* produce OMVs to induce cytotoxicity of epithelial cells [104]. These evidences clearly show cell injury and decreasing amounts of cells during the period of bacterial infection, which may promote the spread of bacteria and damage host defense system against infected bacteria.

## Application of bacterial OMVs as vaccines

OMVs possess several inherent characteristics that make them consider as a potential vaccine candidate. (1) OMVs remain intact and steady at different temperatures and treatments [18, 21, 105]. Considering the properties, OMVs are designed as storage containers to protect enzymatic function [106]. (2) they have inanimate activity, non-replicative property and adjuvant effect [105], but they contain substantial immunogenic components [1] associated with parent bacteria, which may elicit potent innate and adaptive immune responses against bacterial infection [73–79]. Shiga toxin-producing *Escherichia coli* (STEC) OMVs can be treated as vaccines to protect mice and claves against Hemolytic Uremic Syndrome (HUS) [106]. OMVs derived from *Bordetella bronchiseptica* can survive mice from sublethal infection [107]. *Salmonella enteritidis* OMVs provide strong protective efficiency against *S. enteritidis* infection [108]. In addition, OMVs are multipurpose so that they can be engineered as vehicles to carry any antigens or anything else. Yoshihiro Ojima et al.*,* packaged green fluorescence protein (GFP) into the OMVs of *Escherichia coli* and succeed to express OmpW-GFP recombinant protein in OMVs [109]. Engineer OMVs deliver surface-associated glycotopes to the immune system and then induce protective antibody [110]. *Neisseria meningitidis* OMVs modified by genetic technique show stronger immune response than native OMVs [111]. Moreover, they can be modified by special technology to reduce their toxicity and become safe to host. To our knowledge, OMV-based meningococcal vaccine is the only implication of OMVs for clinical trial to control a clonal B outbreak so far [112, 113]. However, because vaccines derived from OMVs are infancy and strategies are immature (shown in Table 2). Vaccines made of OMVs require more time and efforts to be licensed for human use even if amounts of researches have proved their efficiency in animal model (shown in Table 3).

**Table 2:** Limitation of vaccines based on bacterial OMVs

Parent bacteria	Limitation	Resolvent	References
STEC strain (O157:H7 serotype)	Efficiency restricted to O157:H7 serotype	multi-antigenicity vaccines need to be explored	85
*S. Enteritidis*	Slightly toxicity	Improved stratgies to reduce endotoxin activity	87
*E. coli*	verification of protection effects only in mice	Further evaluation on other species	93
*Neisseria meningitidis*	Short duration when immunization beyond the vaccine strain	Repeat immunization and adapt other components of vaccines to OMVs	91
*Neisseria meningitis*	the safety need to be considered because of potent immune response	Vaccination with aluminum hydroxide to ensure safety	95
*V. cholerae*	LPS endotoxicity and expensive production of OMVs	Recombinant OMVs to reduce endotoxicity and advanced methods on OMVs isolation	99

Abbreviations: *STEC*, Shiga toxin-producing Escherichia coli; *OMVs*, outer membrane vesicles; *S. Enteritidis*, Salmonella Enteritidis; *E.coli*, Escherichia coli; *V. cholerae*, Vibrio cholerae; *LPS*, lipopolysaccharide;

**Table 3:** Evaluations of immune protective response mediated by bacterial OMVs against infection in mice model

Parent bacteria	Model establishment	OMVs administration	Adjuvant used	Efficiency	Immune response	References
*E. coli*	porcine pleuropneumonia	subcutaneous	aluminum	87.5% survival for APP serotype 7	Serum IgG Th1 and Th2 cytokines secretion	93
				62.5% survival for APP serotype 1		
S. Enteritidis	foodborne infections of S. Enteritidis	intranasal or intraperitoneal	none	83.3% survival intranasally	Serum IgG and secretory IgA	87
				91% survival intraperitoneally		
Salmonella enterica	Oral infection	Intranasally or intraperitoneally	none	100% survival by Intraperitoneal immunization	Serum IgG and mucosal IgA	94
				80% survival by intranasal immunization		
Neisseria meningitis	Infection	subcutaneous	aluminum hydroxide	100% survival	Serum IgG	95
Neisseria meningitidis	Healthy neonatal mice	intranasal and subcutaneous	DODAB-BF and aluminum hydroxide	Not mentioned	IgG, Intranasal immunization, Th1 and Th2 response,	96
					Th1 profile for subcutaneous immunization	
P. gingivalis	Infection	intranasal	Poly (I:C)	Not mentioned	Serum IgG (including IgG1 and IgG2a) salivary S-IgA	84
*K. pneumoniae*	Sepsis	intraperitoneal	none	80% survival with 0.5 μg OMVs and 100% survival with 1 μg OMVs	Serum IgG and the secretion of key cytokines of Th1 cells (IFN-γ)	62
B. pseudomallei	Septicemic infection	subcutaneous	none	100% survival compared with40% survival in naive mice	serum IgG (IgG1, IgG2a, and IgG3) and IgM	97
Bordetella pertussis	Infection	intraperitoneal	none	Not mentioned	serum IgG, Th1 and Th17 response (the classic whole cell vaccine)	98
					Th1/Th17 and Th2 mixed response (acellular vaccines)	
V. cholerae	Infection	Oral immunization	none	> 80% protection	serum IgG, IgA, IgM, mucosal sIgA, Th2 and Th17 cell response	99
Shigella boydii	Infection	Oral immunization	none	100% protection	mucosal IgG and IgA, Th1 cell response	54
*B. abortus*	Infection	subcutaneous	Freund’s complete and incomplete adjuvant	Not mentioned	Serum IgG, cellular immune response	100

Abbreviations: *E.coli*, Escherichia coli; *APP*, Actinobacillus pleuropneumoniae; *S. Enteritidis*, Salmonella Enteritidis; *Th*, T helper;*P. gingivalis*, Porphyromonas gingivalis;*Ig*, immunoglobulin; *K. pneumoniae*, *Klebsiella pneumoniae*;*B. pseudomallei*, Burkholderia pseudomallei; *V. cholerae*, Vibrio cholerae; *B. abortus*, Brucella abortus; Infection means mice challenged with the same bacteria if not specific statement

## Conclusion

**Fig. 3:**
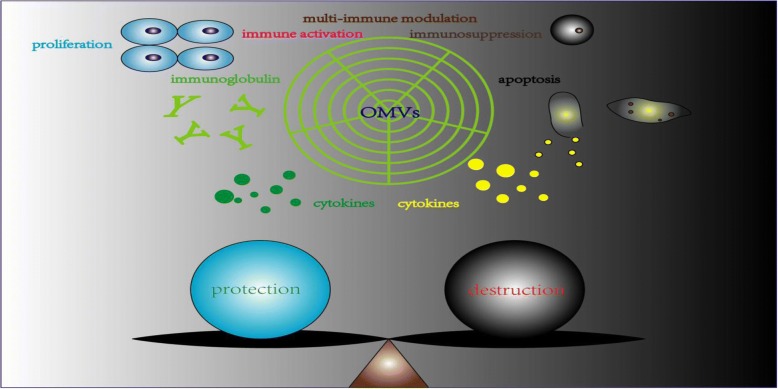
Double-edged effects of OMVs in host. Legend section: protection; destruction; proliferation; immunoglobulin; cytokines; immune activation; immunosuppression; apoptosis

Bacterial OMVs were discovered almost half a century ago, scientists have never desisted from exploring the mechanisms and effects about OMVs. According to the recent study, despite of obscure mechanisms regarding to OMVs formation, we have a better understanding of the mechanisms of OMVs entry, which may enlighten us of how OMVs communicate with host cells. However, a mode of OMVs entry cannot be adopted by all bacteria and we wonder if there is a common way that OMVs can take. Plenty of evidences elucidate the significant effects of OMVs on host cells. We can summarize that OMVs can drive host cells to produce more pro-inflammatory and anti-inflammatory cytokines in response to OMVs challenge. Meanwhile, innate and adaptive immune response can be imitated by exposure to OMVs. In contrast, OMVs have the capacity of inhibiting immune cells responses [69, 85] which may contribute to bacteria survival and spread. It seems that OMVs can play dual role in modulating immune system response against infection (shown in Fig. 3). Together with the responses caused by OMVs, OMVs may play an important role in bacterial pathogenesis and the interactions between pathogen and host cells. There is one report regarding OMVs in indoor dust elucidating the association between asthma with OMVs, which may remind us that OMVs may have a broader effect on inflammatory [114].

Furthermore, OMV-based vaccines show great potential in the development of immunity to diseases caused by bacteria. Innovative technologies make bioengineered OMVs possible to serve as specific vaccines targeted on limited pathogen [115]. Enlightened from the technologies, scientists utilize modified OMVs just as a vehicle to carry drugs or anything else if they want to evaluate the effects on other diseases even cancer, which broad the therapeutic implications and expand the field of OMVs applications, not just restricted to bacterial infection. For instance, bacterial OMVs are engineered as multi-functional nanodevices for biosensing and bioimaging of cancer cells [116]. OMVs decorated with tumor-specific epitopes can induce strong inhibition of tumor growth [117]. Collectively, advances are made to expand our knowledge of exploiting OMVs-associated techniques to benefit the health of human worldwide.

## Abbreviations

ABC: ATP-binding cassette
APCs: Antigen presenting cells
*B. fragilis*: *Bacteroides fragilis*
BCR: B cell receptor
BMDM: Bone marrow-derived macrophages
BPI: Bactericidal/permeability-increasing protein
CD: Cluster Of Differentiation
CNF1: Cytotoxic necrotizing factor type 1
GBP: Guanylate Binding Proteins
GFP: Green fluorescence protein
HLA: Human leukocyte antigen
HUS: Hemolytic Uremic Syndrome
ICAM: Intercellular adhesion molecules
IFN-γ: Gamma interferon
Ig: Immunoglobulin
IL: Interleukin
*L. pneumophila*: *Legionella pneumophila*
LPS: Lipopolysaccharide
MHC: Major histocompatibility complex
MIP: Macrophage inflammatory protein
NETs: Neutrophil extracellular traps
OMVs: Outer membrane vesicles
*P. gingivalis*: *Porphyromonas gingivalis*
PAMPs: Pathogen associated molecular patterns
PBMC: Peripheral blood mononuclear cells
PD-L1: Programmed death-ligand 1
PMNs: Polymorphonuclear leukocytes
PQS: Pseudomonas Quinolone Signal
STEC: Shiga toxin-producing *Escherichia coli*
Th: T helper cell
TLR: Toll like receptor
TNF-α: Tumor necrosis factor alpha
UPEC: Uropathogenic *Escherichia coli*

## References

[1] Gasperini GBM, Arato V, Gianfaldoni C, Vadi A, Norais N, Bensi G, Delany IPM, Aricò B, Leuzzi R (2018). Outer Membrane Vesicles (OMV)-based and Proteomics-driven Antigen Selection Identifies Novel Factors Contributing to Bordetella pertussis Adhesion to Epithelial Cells. Mol Cell Proteomics..

[2] Kothary MH, Gopinath GR, Gangiredla J, Rallabhandi PV, Harrison LM, Yan QQ (2017). Analysis and characterization of proteins associated with outer membrane vesicles secreted by Cronobacter spp. Front Microbiol.

[3] McCaig WD, Loving CL, Hughes HR, Brockmeier SL (2016). Characterization and vaccine potential of outer membrane vesicles produced by Haemophilus parasuis. PLoS One.

[4] Fulsundar S, Kulkarni HM, Jagannadham MV, Nair R, Keerthi S, Sant P (2015). Molecular characterization of outer membrane vesicles released from Acinetobacter radioresistens and their potential roles in pathogenesis. Microb Pathog..

[5] Bitto NJ, Chapman R, Pidot S, Costin A, Lo C, Choi J (2017). Bacterial membrane vesicles transport their DNA cargo into host cells. Sci Rep.

[6] Koeppen K, Hampton TH, Jarek M, Scharfe M, Gerber SA, Mielcarz DW (2016). A novel mechanism of host-pathogen interaction through sRNA in bacterial outer membrane vesicles. PLoS Pathog.

[7] Choi JW, Kim SC, Hong SH, Lee HJ (2017). Secretable small RNAs via outer membrane vesicles in periodontal pathogens. J Dent Res.

[8] Aschtgen MS, Lynch JB, Koch E, Schwartzman J, McFall-Ngai M, Ruby E (2016). Rotation of Vibrio fischeri flagella produces outer membrane vesicles that induce host development. J Bacteriol.

[9] Arntzen MO, Varnai A, Mackie RI, Eijsink VGH, Pope PB (2017). Outer membrane vesicles from Fibrobacter succinogenes S85 contain an array of carbohydrate-active enzymes with versatile polysaccharide-degrading capacity. Environ Microbiol.

[10] Li J, Azam F, Zhang S (2016). Outer membrane vesicles containing signalling molecules and active hydrolytic enzymes released by a coral pathogen Vibrio shilonii AK1. Environ Microbiol.

[11] DA Thay B, Kufer TA, Wai SN, Oscarsson J (2014). Aggregatibacter. actinomycetemcomitans outer membrane vesicles are internalized in human host cells and trigger NOD1- and NOD2-dependent NF-κB activation. Infect Immun..

[12] Kaparakis-Liaskos M, Ferrero RL (2015). Immune modulation by bacterial outer membrane vesicles. Nat Rev Immunol.

[13] Hickey CA, Kuhn KA, Donermeyer DL, Porter NT, Jin C, Cameron EA (2015). Colitogenic Bacteroides thetaiotaomicron antigens access host immune cells in a sulfatase-dependent manner via outer membrane vesicles. Cell Host Microbe.

[14] Vanhove AS, Duperthuy M, Charriere GM, Le Roux F, Goudenege D, Gourbal B (2015). Outer membrane vesicles are vehicles for the delivery of Vibrio tasmaniensis virulence factors to oyster immune cells. Environ Microbiol.

[15] Chatterjee S, Mondal A, Mitra S, Basu S (2017). Acinetobacter baumannii transfers the blaNDM-1 gene via outer membrane vesicles. J Antimicrob Chemother.

[16] Stentz R, Horn N, Cross K, Salt L, Brearley C, Livermore DM (2015). Cephalosporinases associated with outer membrane vesicles released by Bacteroides spp. protect gut pathogens and commensals against beta-lactam antibiotics. J Antimicrob Chemother.

[17] Brown L, Kessler A, Cabezas-Sanchez P, Luque-Garcia JL, Casadevall A (2014). Extracellular vesicles produced by the gram-positive bacterium Bacillus subtilis are disrupted by the lipopeptide surfactin. Mol Microbiol.

[18] Bauwens A, Kunsmann L, Marejkova M, Zhang W, Karch H, Bielaszewska M (2017). Intrahost milieu modulates production of outer membrane vesicles, vesicle-associated Shiga toxin 2a and cytotoxicity in Escherichia coli O157:H7 and O104:H4. Environ Microbiol Rep.

[19] Chen Y, Liu L, Fu H, Wei C, Jin Q (2014). Comparative proteomic analysis of outer membrane vesicles from Shigella flexneri under different culture conditions. Biochem Biophys Res Commun.

[20] Turner L, Bitto NJ, Steer DL, Lo C, D'Costa K, Ramm G (2018). Helicobacter pylori outer membrane vesicle size determines their mechanisms of host cell entry and protein content. Front Immunol.

[21] Adriani R, Mousavi Gargari SL, Nazarian S, Sarvary S, Noroozi N (2018). Immunogenicity of Vibrio cholerae outer membrane vesicles secreted at various environmental conditions. Vaccine.

[22] Devos S, Van Putte W, Vitse J, Van Driessche G, Stremersch S, Van Den Broek W (2017). Membrane vesicle secretion and prophage induction in multidrug-resistant Stenotrophomonas maltophilia in response to ciprofloxacin stress. Environ Microbiol.

[23] Bauwens AKL, Karch H, Mellmann A, Bielaszewska M. Antibiotic-Mediated Modulations of Outer Membrane Vesicles in Enterohemorrhagic *Escherichia coli* O104:H4 and O157:H7. Antimicrob Agents Chemother. 2017;61(9). 10.1128/AAC.00937-17 PMC5571282 28607018

[24] Gamalier JP, Silva TP, Zarantonello V, Dias FF, Melo RC. Increased production of outer membrane vesicles by cultured freshwater bacteria in response to ultraviolet radiation. Microbiol Res. 2017;194:38–46. 10.1016/j.micres.2016.08.002. 10.1016/j.micres.2016.08.002 27938861

[25] Marisa Heredia R, Sabrina Boeris P, Sebastian Liffourrena A, Fernanda Bergero M, Alberto Lopez G, Ines LG (2016). Release of outer membrane vesicles in Pseudomonas putida as a response to stress caused by cationic surfactants. Microbiology.

[26] Kim SW, Park SB, Im SP, Lee JS, Jung JW, Gong TW (2018). Outer membrane vesicles from beta-lactam-resistant *Escherichia coli* enable the survival of beta-lactam-susceptible *E. coli* in the presence of beta-lactam antibiotics. Sci Rep..

[27] Urashima A, Sanou A, Yen H, Tobe T. Enterohaemorrhagic *Escherichia coli* produces outer membrane vesicles as an active defence system against antimicrobial peptide LL-37. Cell Microbiol. 2017;19(11). 10.1111/cmi.12758 28622430

[28] Reyes-Robles T, Dillard RS, Cairns LS, Silva-Valenzuela CA, Housman M, Ali A, et al. Vibrio cholerae outer membrane vesicles inhibit bacteriophage infection. J Bacteriol. 2018. 10.1128/JB.00792-17 PMC6040182 29661863

[29] Waller T, Kesper L, Hirschfeld J, Dommisch H, Kolpin J, Oldenburg J, et al. Porphyromonas gingivalis outer membrane vesicles induce selective tumor necrosis factor tolerance in a toll-like receptor 4- and mTOR-dependent manner. Infect Immun. 2016;84(4):1194–204. 10.1128/IAI.01390-15. 10.1128/IAI.01390-15 PMC4807478 26857578

[30] Cahill BK, Seeley KW, Gutel D, Ellis TN. Klebsiella pneumoniae O antigen loss alters the outer membrane protein composition and the selective packaging of proteins into secreted outer membrane vesicles. Microbiol Res. 2015;180:1–10. 10.1016/j.micres.2015.06.012. 10.1016/j.micres.2015.06.012 26505306

[31] Murase KMP, Porcheron G, Houle S, Helloin E, Pénary M, Nougayrède JP, Dozois CM, Hayashi T, Oswald E (2016). HlyF Produced by Extraintestinal Pathogenic *Escherichia coli* Is a Virulence Factor That Regulates Outer Membrane Vesicle Biogenesis. J Infect Dis..

[32] Roier S, Zingl FG, Cakar F, Durakovic S, Kohl P, Eichmann TO (2016). A novel mechanism for the biogenesis of outer membrane vesicles in gram-negative bacteria. Nat Commun.

[33] Florez C, Raab JE, Cooke AC, Schertzer JW (2017). Membrane Distribution of the Pseudomonas Quinolone Signal Modulates Outer Membrane Vesicle Production in Pseudomonas aeruginosa. MBio..

[34] Horspool AM, Schertzer JW (2018). Reciprocal cross-species induction of outer membrane vesicle biogenesis via secreted factors. Sci Rep.

[35] Hampton CM, Guerrero-Ferreira RC, Storms RE, Taylor JV, Yi H, Gulig PA (2017). The opportunistic pathogen Vibrio vulnificus produces outer membrane vesicles in a spatially distinct manner related to capsular polysaccharide. Front Microbiol.

[36] Premjani V, Tilley D, Gruenheid S, Le Moual H, Samis JA (2014). Enterohemorrhagic Escherichia coli OmpT regulates outer membrane vesicle biogenesis. FEMS Microbiol Lett.

[37] Zakharzhevskaya NB, Vanyushkina AA, Altukhov IA, Shavarda AL, Butenko IO, Rakitina DV (2017). Outer membrane vesicles secreted by pathogenic and nonpathogenic Bacteroides fragilis represent different metabolic activities. Sci Rep.

[38] Elhenawy W, Bording-Jorgensen M, Valguarnera E, Haurat MF, Wine E, Feldman MF (2016). LPS Remodeling Triggers Formation of Outer Membrane Vesicles in Salmonella. MBio..

[39] Bonnington KE, Kuehn MJ (2016). Outer Membrane Vesicle Production Facilitates LPS Remodeling and Outer Membrane Maintenance in Salmonella during Environmental Transitions. MBio..

[40] Nakayama-Imaohji H, Hirota K, Yamasaki H, Yoneda S, Nariya H, Suzuki M (2016). DNA inversion regulates outer membrane vesicle production in Bacteroides fragilis. PLoS One.

[41] Choi HI, Kim M, Jeon J, Han JK, Kim KS (2017). Overexpression of MicA induces production of OmpC-enriched outer membrane vesicles that protect against Salmonella challenge. Biochem Biophys Res Commun.

[42] Canas MA, Gimenez R, Fabrega MJ, Toloza L, Baldoma L, Badia J (2016). Outer membrane vesicles from the probiotic Escherichia coli Nissle 1917 and the commensal ECOR12 enter intestinal epithelial cells via Clathrin-dependent endocytosis and elicit differential effects on DNA damage. PLoS One.

[43] Kunsmann L, Ruter C, Bauwens A, Greune L, Gluder M, Kemper B (2015). Virulence from vesicles: novel mechanisms of host cell injury by Escherichia coli O104:H4 outbreak strain. Sci Rep.

[44] Vanaja SK, Russo AJ, Behl B, Banerjee I, Yankova M, Deshmukh SD (2016). Bacterial outer membrane vesicles mediate cytosolic localization of LPS and Caspase-11 activation. Cell.

[45] O'Donoghue EJ, Sirisaengtaksin N, Browning DF, Bielska E, Hadis M, Fernandez-Trillo F (2017). Lipopolysaccharide structure impacts the entry kinetics of bacterial outer membrane vesicles into host cells. PLoS Pathog.

[46] Gu L, Meng R, Tang Y, Zhao K, Liang F, Zhang R, et al. Toll like receptor 4 signaling licenses the cytosolic transport of lipopolysaccharide from bacterial outer membrane vesicles. Shock. 2018. 10.1097/SHK.0000000000001129. 10.1097/SHK.0000000000001129 29462003

[47] Jager J, Keese S, Roessle M, Steinert M, Schromm AB (2015). Fusion of legionella pneumophila outer membrane vesicles with eukaryotic membrane systems is a mechanism to deliver pathogen factors to host cell membranes. Cell Microbiol.

[48] Fernandez-Rojas MA, Vaca S, Reyes-Lopez M, de la Garza M, Aguilar-Romero F, Zenteno E (2014). Outer membrane vesicles of Pasteurella multocida contain virulence factors. MicrobiologyOpen.

[49] Berleman JE, Allen S, Danielewicz MA, Remis JP, Gorur A, Cunha J (2014). The lethal cargo of Myxococcus xanthus outer membrane vesicles. Front Microbiol.

[50] Zakharzhevskaya NB, Tsvetkov VB, Vanyushkina AA, Varizhuk AM, Rakitina DV, Podgorsky VV (2017). Interaction of Bacteroides fragilis toxin with outer membrane vesicles reveals new mechanism of its secretion and delivery. Front Cell Infect Microbiol.

[51] Valeru SP, Shanan S, Alossimi H, Saeed A, Sandstrom G, Abd H (2014). Lack of outer membrane protein a enhances the release of outer membrane vesicles and survival of Vibrio cholerae and suppresses viability of Acanthamoeba castellanii. Int J Microbiol..

[52] Lapinet JASP, Calzetti F, Pérez O, Cassatella MA (2000). Gene expression and production of tumor necrosis factor alpha, interleukin-1beta (IL-1beta), IL-8, macrophage inflammatory protein 1alpha (MIP-1alpha), MIP-1beta, and gamma interferon-inducible protein 10 by human neutrophils stimulated with group B meningococcal outer membrane vesicles. Infect Immun..

[53] Davis JM, Carvalho HM, Rasmussen SB, O'Brien AD (2006). Cytotoxic necrotizing factor type 1 delivered by outer membrane vesicles of uropathogenic Escherichia coli attenuates polymorphonuclear leukocyte antimicrobial activity and chemotaxis. Infect Immun.

[54] Vida A, Troelstra A, Antal-Szalmas P, van Bommel TJ, Verheul AF, Verhoef J (2011). Neutralization of Neisseria meningitidis outer membrane vesicles. Inflamm Res..

[55] Pilsczek FH, Salina D, Poon KK, Fahey C, Yipp BG, Sibley CD (2010). A novel mechanism of rapid nuclear neutrophil extracellular trap formation in response to Staphylococcus aureus. J Immunol.

[56] Lappann M, Danhof S, Guenther F, Olivares-Florez S, Mordhorst IL, Vogel U (2013). In vitro resistance mechanisms of Neisseria meningitidis against neutrophil extracellular traps. Mol Microbiol.

[57] Gao XJ, Li T, Wei B, Yan ZX, Hu N, Huang YJ (2018). Bacterial outer membrane vesicles from dextran sulfate sodium-induced colitis differentially regulate intestinal UDP-glucuronosyltransferase 1A1 partially through toll-like receptor 4/mitogen-activated protein kinase/phosphatidylinositol 3-kinase pathway. Drug Metab Dispos..

[58] Mitra S, Sinha R, Mitobe J, Koley H (2016). Development of a cost-effective vaccine candidate with outer membrane vesicles of a tolA-disrupted Shigella boydii strain. Vaccine.

[59] Alaniz RC, Deatherage BL, Lara JC, Cookson BT (2007). Membrane vesicles are immunogenic facsimiles of Salmonella typhimurium that potently activate dendritic cells, prime B and T cell responses, and stimulate protective immunity in vivo. J Immunol.

[60] Cecil JD, O'Brien-Simpson NM, Lenzo JC, Holden JA, Singleton W, Perez-Gonzalez A (2017). Outer membrane vesicles prime and activate macrophage Inflammasomes and cytokine secretion in vitro and in vivo. Front Immunol.

[61] Fleetwood AJ, Lee MKS, Singleton W, Achuthan A, Lee MC, O'Brien-Simpson NM (2017). Metabolic remodeling, Inflammasome activation, and Pyroptosis in macrophages stimulated by Porphyromonas gingivalis and its outer membrane vesicles. Front Cell Infect Microbiol.

[62] Jung AL, Hoffmann K, Herkt CE, Schulz C, Bertrams W, Schmeck B. Legionella pneumophila outer membrane vesicles: isolation and analysis of their pro-inflammatory potential on macrophages. J Vis Exp. 2017;120.e55146. 10.3791/55146. 10.3791/55146 PMC5409326 28287548

[63] Jung AL, Stoiber C, Herkt CE, Schulz C, Bertrams W, Schmeck B (2016). Legionella pneumophila-derived outer membrane vesicles promote bacterial replication in macrophages. PLoS Pathog.

[64] Finethy RLS, Orench-Rivera N, Feeley EM, Haldar AK, Yamamoto M, Kanneganti TD, Kuehn MJ, Coers J (2017). Inflammasome Activation by Bacterial Outer Membrane Vesicles Requires Guanylate Binding Proteins. MBio..

[65] Turner KL, Cahill BK, Dilello SK, Gutel D, Brunson DN, Alberti S (2015). Porin loss impacts the host inflammatory response to outer membrane vesicles of Klebsiella pneumoniae. Antimicrob Agents Chemother.

[66] Tavano R, Franzoso S, Cecchini P, Cartocci E, Oriente F, Arico B (2009). The membrane expression of Neisseria meningitidis adhesin a (NadA) increases the proimmune effects of MenB OMVs on human macrophages, compared with NadA- OMVs, without further stimulating their proinflammatory activity on circulating monocytes. J Leukoc Biol.

[67] Lee WH, Choi HI, Hong SW, Kim KS, Gho YS, Jeon SG (2015). Vaccination with Klebsiella pneumoniae-derived extracellular vesicles protects against bacteria-induced lethality via both humoral and cellular immunity. Exp Mol Med.

[68] Deo P, Chow SH, Hay ID, Kleifeld O, Costin A, Elgass KD (2018). Outer membrane vesicles from Neisseria gonorrhoeae target PorB to mitochondria and induce apoptosis. PLoS Pathog.

[69] Winter JLD, Rhead J, Atherton J, Robinson K (2014). Helicobacter pylori membrane vesicles stimulate innate pro- and anti-inflammatory responses and induce apoptosis in Jurkat T cells. Infect Immun..

[70] Laughlin RC, Mickum M, Rowin K, Adams LG, Alaniz RC (2015). Altered host immune responses to membrane vesicles from Salmonella and gram-negative pathogens. Vaccine.

[71] Zariri A, Beskers J, van de Waterbeemd B, Hamstra HJ, Bindels TH, van Riet E (2016). Meningococcal outer membrane vesicle composition-dependent activation of the innate immune response. Infect Immun.

[72] Ko SH, Rho DJ, Jeon JI, Kim YJ, Woo HA, Kim N (2016). Crude preparations of helicobacter pylori outer membrane vesicles induce upregulation of Heme Oxygenase-1 via activating Akt-Nrf2 and mTOR-IkappaB kinase-NF-kappaB pathways in dendritic cells. Infect Immun.

[73] Liu Q, Liu Q, Yi J, Liang K, Liu T, Roland KL (2016). Outer membrane vesicles derived from Salmonella typhimurium mutants with truncated LPS induce cross-protective immune responses against infection of Salmonella enterica serovars in the mouse model. Int J Med Microbiol..

[74] Tunheim G, Arnemo M, Naess LM, Norheim G, Garcia L, Cardoso D (2016). Immune responses of a meningococcal a + W outer membrane vesicle (OMV) vaccine with and without aluminium hydroxide adjuvant in two different mouse strains. APMIS..

[75] de Oliveira Santos FA, Lincopan N, De Gaspari E (2018). Evaluation of intranasal and subcutaneous route of immunization in neonatal mice using DODAB-BF as adjuvant with outer membrane vesicles of Neisseria meningitis B. Immunobiology..

[76] Nieves WPH, Judy BM, Blumentritt CA, Russell-Lodrigue K, Roy CJ, Torres AGML (2014). A Burkholderia pseudomallei outer membrane vesicle vaccine provides protection against lethal sepsis. Clin Vaccine Immunol..

[77] Bottero D, Gaillard ME, Zurita E, Moreno G, Martinez DS, Bartel E (2016). Characterization of the immune response induced by pertussis OMVs-based vaccine. Vaccine.

[78] Sinha R, Koley H, Nag D, Mitra S, Mukhopadhyay AK, Chattopadhyay B (2015). Pentavalent outer membrane vesicles of Vibrio cholerae induce adaptive immune response and protective efficacy in both adult and passive suckling mice models. Microbes Infect.

[79] Kaur G, Singh S, Sunil Kumar BV, Mahajan K, Verma R (2016). Characterization and immunogenicity of outer membrane vesicles from Brucella abortus. J Immunoassay Immunochem..

[80] Vaughan AT, Brackenbury LS, Massari P, Davenport V, Gorringe A, Heyderman RS (2010). Neisseria lactamica selectively induces mitogenic proliferation of the naive B cell pool via cell surface Ig. J Immunol.

[81] Vaughan AT, Gorringe A, Davenport V, Williams NA, Heyderman RS (2009). Absence of mucosal immunity in the human upper respiratory tract to the commensal bacteria Neisseria lactamica but not pathogenic Neisseria meningitidis during the peak age of nasopharyngeal carriage. J Immunol.

[82] Stevenson FK, Perez Vidakovics MLA, Jendholm J, Mörgelin M, Månsson A, Larsson C (2010). B cell activation by outer membrane vesicles—a novel virulence mechanism. PLoS Pathog.

[83] Lee DH, Kim S-H, Kang W, Choi YS, Lee S-H, Lee S-R (2011). Adjuvant effect of bacterial outer membrane vesicles with penta-acylated lipopolysaccharide on antigen-specific T cell priming. Vaccine.

[84] Youssef AR, van der Flier M, Estevao S, Hartwig NG, van der Ley P, Virji M (2009). Opa+ and Opa- isolates of Neisseria meningitidis and Neisseria gonorrhoeae induce sustained proliferative responses in human CD4+ T cells. Infect Immun.

[85] Hock BD, McKenzie JL, Keenan JI. Helicobacter pylori outer membrane vesicles inhibit human T cell responses via induction of monocyte COX-2 expression. Pathog Dis. 2017;75(4). 10.1093/femspd/ftx034. 10.1093/femspd/ftx034 28430970

[86] Zhu W, Tomberg J, Knilans KJ, Anderson JE, McKinnon KP, Sempowski GD (2018). Properly folded and functional PorB fromNeisseria gonorrhoeaeinhibits dendritic cell stimulation of CD4+T cell proliferation. J Biol Chem.

[87] Rasti ES, Schappert ML, Brown AC (2018). Association of Vibrio cholerae 569B outer membrane vesicles with host cells occurs in a GM1-independent manner. Cell Microbiol.

[88] Aschtgen MS, Wetzel K, Goldman W, McFall-Ngai M, Ruby E (2016). Vibrio fischeri-derived outer membrane vesicles trigger host development. Cell Microbiol.

[89] Kim YJJH, Na SH, Kwon HI, Selasi GN, Nicholas A, Park TI, Lee SH, Lee JC (2016). Stenotrophomonas maltophilia outer membrane vesicles elicit a potent inflammatory response in vitro and in vivo. Pathog Dis..

[90] Mondal A, Tapader R, Chatterjee NS, Ghosh A, Sinha R, Koley H (2016). Cytotoxic and inflammatory responses induced by outer membrane vesicle-associated biologically active proteases from Vibrio cholerae. Infect Immun.

[91] Bitto NJ, Baker PJ, Dowling JK, Wray-McCann G, De Paoli A, Tran LS, et al. Membrane vesicles from Pseudomonas aeruginosa activate the non-canonical inflammasome through caspase-5 in human monocytes. Immunol Cell Biol. 2018. 10.1111/imcb.12190. 10.1111/imcb.12190 30003588

[92] Canas MA, Fabrega MJ, Gimenez R, Badia J, Baldoma L (2018). Outer membrane vesicles from probiotic and commensal Escherichia coli activate NOD1-mediated immune responses in intestinal epithelial cells. Front Microbiol.

[93] Bielaszewska M, Marejkova M, Bauwens A, Kunsmann-Prokscha L, Mellmann A, Karch H (2018). Enterohemorrhagic Escherichia coli O157 outer membrane vesicles induce interleukin 8 production in human intestinal epithelial cells by signaling via toll-like receptors TLR4 and TLR5 and activation of the nuclear factor NF-kappaB. Int J Med Microbiol..

[94] Jha C, Ghosh S, Gautam V, Malhotra P, Ray P (2017). In vitro study of virulence potential of Acinetobacter baumannii outer membrane vesicles. Microb Pathog.

[95] Ko SH, Jeon JI, Kim YJ, Yoon HJ, Kim H, Kim N (2015). Helicobacter pylori outer membrane vesicle proteins induce human eosinophil degranulation via a beta2 integrin CD11/CD18- and ICAM-1-dependent mechanism. Mediat Inflamm.

[96] Yang WW, Guo B, Jia WY, Jia Y (2016). Porphyromonas gingivalis-derived outer membrane vesicles promote calcification of vascular smooth muscle cells through ERK1/2-RUNX2. FEBS open bio.

[97] Ho MH, Guo ZM, Chunga J, Goodwin JS, Xie H (2016). Characterization of innate immune responses of human endothelial cells induced by Porphyromonas gingivalis and their derived outer membrane vesicles. Front Cell Infect Microbiol.

[98] Alvarez CS, Badia J, Bosch M, Gimenez R, Baldoma L (2016). Outer membrane vesicles and soluble factors released by probiotic Escherichia coli Nissle 1917 and commensal ECOR63 enhance barrier function by regulating expression of tight junction proteins in intestinal epithelial cells. Front Microbiol.

[99] Kim JH, Yoon YJ, Lee J, Choi EJ, Yi N, Park KS (2013). Outer membrane vesicles derived from Escherichia coli up-regulate expression of endothelial cell adhesion molecules in vitro and in vivo. PLoS One.

[100] Metruccio MM, Evans DJ, Gabriel MM, Kadurugamuwa JL, Fleiszig SM (2016). Pseudomonas aeruginosa outer membrane vesicles triggered by human mucosal fluid and lysozyme can prime host tissue surfaces for bacterial adhesion. Front Microbiol.

[101] Elmi A, Nasher F, Jagatia H, Gundogdu O, Bajaj-Elliott M, Wren B (2016). Campylobacter jejuni outer membrane vesicle-associated proteolytic activity promotes bacterial invasion by mediating cleavage of intestinal epithelial cell E-cadherin and occludin. Cell Microbiol.

[102] Bielaszewska M, Ruter C, Bauwens A, Greune L, Jarosch KA, Steil D (2017). Host cell interactions of outer membrane vesicle-associated virulence factors of enterohemorrhagic Escherichia coli O157: intracellular delivery, trafficking and mechanisms of cell injury. PLoS Pathog.

[103] Rumbo C, Tomas M, Fernandez Moreira E, Soares NC, Carvajal M, Santillana E (2014). The Acinetobacter baumannii Omp33-36 porin is a virulence factor that induces apoptosis and modulates autophagy in human cells. Infect Immun.

[104] Nho JS, Jun SH, Oh MH, Park TI, Choi CW, Kim SI (2015). Acinetobacter nosocomialis secretes outer membrane vesicles that induce epithelial cell death and host inflammatory responses. Microb Pathog.

[105] Nakao R, Hasegawa H, Dongying B, Ohnishi M, Senpuku H (2016). Assessment of outer membrane vesicles of periodontopathic bacterium Porphyromonas gingivalis as possible mucosal immunogen. Vaccine.

[106] Fingermann M, Avila L, De Marco MB, Vazquez L, Di Biase DN, Muller AV (2018). OMV-based vaccine formulations against Shiga toxin producing Escherichia coli strains are both protective in mice and immunogenic in calves. Hum Vaccin Immunother..

[107] Bottero D, Zurita ME, Gaillard ME, Bartel E, Vercellini C, Hozbor D (2018). Membrane Vesicles Derived from Bordetella bronchiseptica: Active Constituent of a New Vaccine against Infections Caused by This Pathogen. Appl Environ Microbiol..

[108] Liu Q, Yi J, Liang K, Zhang X, Liu Q (2017). Outer membrane vesicles derived from Salmonella Enteritidis protect against the virulent wild-type strain infection in a mouse model. J Microbiol Biotechnol.

[109] Ojima Y, Yamaguchi K, Taya M (2018). Quantitative evaluation of recombinant protein packaged into outer membrane vesicles of Escherichia coli cells. Biotechnol Prog.

[110] Chen L, Valentine JL, Huang CJ, Endicott CE, Moeller TD, Rasmussen JA (2016). Outer membrane vesicles displaying engineered glycotopes elicit protective antibodies. Proc Natl Acad Sci U S A.

[111] Zhang L, Wen Z, Lin J, Xu H, Herbert P, Wang XM (2016). Improving the immunogenicity of a trivalent Neisseria meningitidis native outer membrane vesicle vaccine by genetic modification. Vaccine.

[112] Sevestre J, Hong E, Delbos V, Terrade A, Mallet E, Deghmane AE (2017). Durability of immunogenicity and strain coverage of MenBvac, a meningococcal vaccine based on outer membrane vesicles: lessons of the Normandy campaign. Vaccine.

[113] Oftung F, Korsvold GE, Aase A, Naess LM (2016). Cellular immune responses in humans induced by two serogroup B meningococcal outer membrane vesicle vaccines given separately and in combination. Clin Vaccine Immunol..

[114] Kim YS, Choi EJ, Lee WH, Choi SJ, Roh TY, Park J (2013). Extracellular vesicles, especially derived from gram-negative bacteria, in indoor dust induce neutrophilic pulmonary inflammation associated with both Th1 and Th17 cell responses. Clin Exp Allergy..

[115] Xu K, Zhao Q, Wen X, Wu R, Wen Y, Huang X (2018). A trivalent Apx-fusion protein delivered by *E. coli* outer membrane vesicles induce protection against Actinobacillus pleuropneumoniae of serotype 1 and 7 challenge in a murine model. PloS one..

[116] Chen QRS, Chen W (2017). Engineering multi-functional bacterial outer membrane vesicles as modular nanodevices for biosensing and bioimaging. Chem Commun (Camb)..

[117] Grandi A, Tomasi M, Zanella I, Ganfini L, Caproni E, Fantappie L (2017). Synergistic protective activity of tumor-specific epitopes engineered in bacterial outer membrane vesicles. Front Oncol.

